# Treatment of periorbital xanthogranuloma with fractional carbon dioxide laser

**DOI:** 10.1016/j.jdcr.2024.03.018

**Published:** 2024-04-08

**Authors:** Travis Benson, Carrie Kovarik, Corbett Berry, Joseph Sobanko, Misha Rosenbach

**Affiliations:** Department of Dermatology, Hospital of the University of Pennsylvania, Philadelphia, Pennsylvania

**Keywords:** CO_2_ laser, fractional ablative laser, fractional photothermolysis, periorbital xanthogranuloma

## Introduction

Adult orbital xanthogranulomatous (XG) disease is a spectrum of granulomatous conditions affecting the orbit and ocular adnexa characterized histologically by the presence of Touton giant cells and foamy histiocytes. This group is comprised of 4 subtypes: necrobiotic xanthogranuloma (NXG), adult-onset xanthogranuloma, adult-onset asthma and periocular xanthogranuloma, and Erdheim-Chester disease.^1^ We present a case of refractory periorbital xanthogranulomas without underlying paraproteinemia with an excellent and durable response to treatment with fractional carbon dioxide (CO_2_) laser.

## Case report

**Fig 1:**
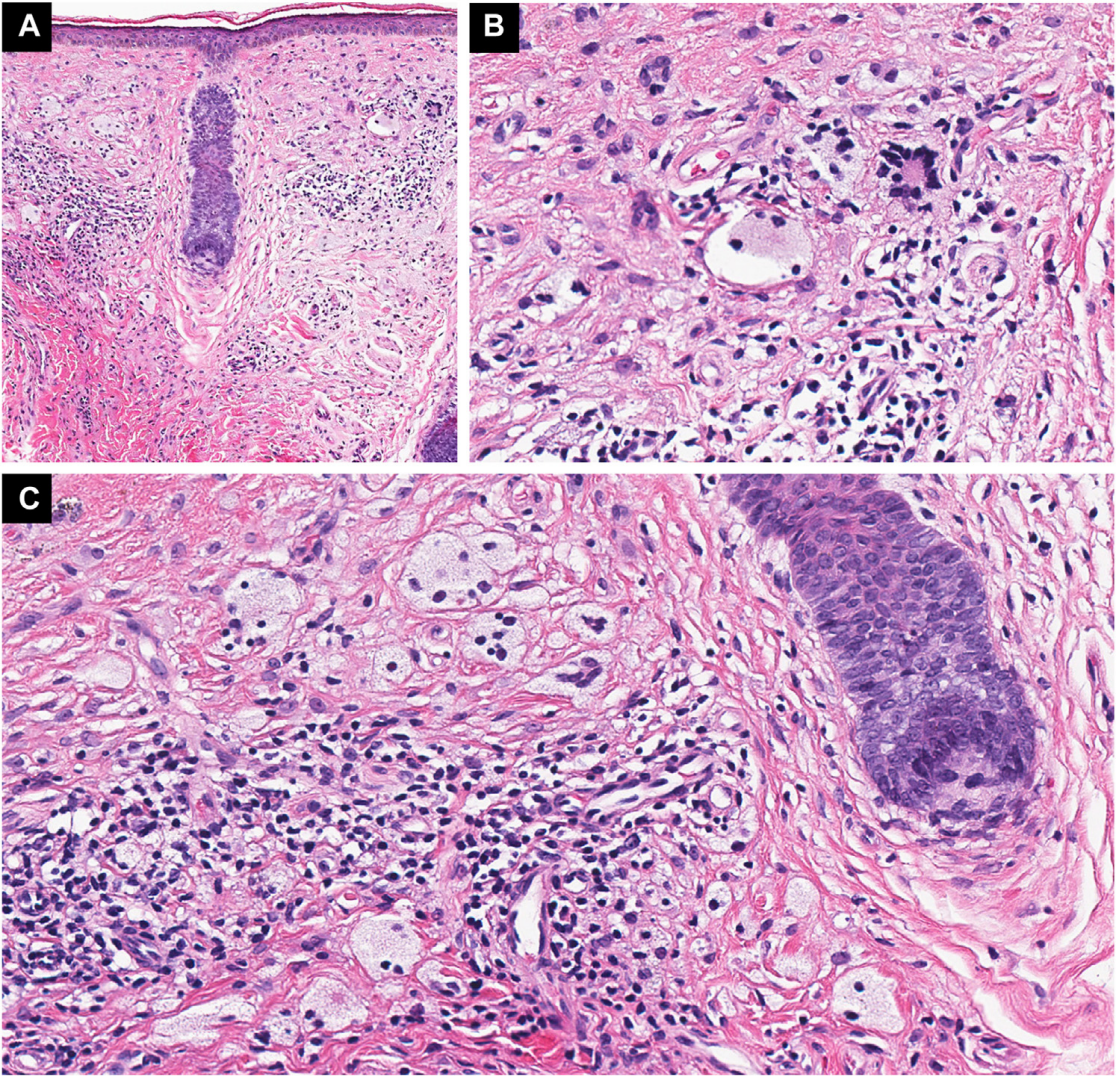
Hematoxylin and eosin (H&E) staining demonstrating xanthomatized histiocytes, Touton giant cells, multinucleated histiocytes, and a lymphocytic infiltrate with rare plasma cells. Magnifications: (**A**) 10×, (**B**) 30×, and (**C**) 30×.

A woman in her 50s presented to dermatology in 2015 for evaluation of discolored plaques on her bilateral cheeks that had been present since 2010. On physical exam, she had yellow-brown hyperpigmented plaques on the bilateral infraorbital cheeks without history of plaque ulceration. A punch biopsy of the left cheek was performed and demonstrated xanthomatized histiocytes, Touton giant cells, multinucleated histiocytes, and a lymphocytic infiltrate with rare plasma cells, consistent with a XG disease. The presence of Touton giant cells and multinucleated histiocytes raised concern for NXG (Fig 1).

The patient was evaluated for paraproteinemia and several subsequent serum protein electrophoresis and immunofixation tests were normal. Over the course of her condition, she had been prescribed triamcinolone 0.025% cream, tretinoin 0.05% cream, clobetasol 0.05% ointment, tacrolimus 0.1% ointment, minocycline, intralesional triamcinolone, hydroxychloroquine, fluocinolone acetonide 0.01%/hydroquinone 4%/tretinoin 0.05% cream, and pentoxifylline, all with minimal efficacy.

In 2019, she was evaluated for treatment of the lesions with Ultrapulse (Lumenis Ltd, Yokneum, Israel) fractional CO_2_ laser (10,600 nm). A test spot of the right cheek was performed to assess risk of pigmentary change using near fully-ablative settings to target the superficial plaques with an energy of 100 mJ, spot size of 8 mm, density of 9%, and a square pattern. The patient responded well and followed up in 2021 for further treatment. Her right cheek was subsequently treated using energy of 100 mJ, power of 1 W, spot size of 8 mm, density of 9%, and a hexagon pattern. The treatment area was anesthetized with 1% lidocaine with epinephrine. Two passes were made to the right cheek with saline debridement performed between passes. A total of 32 pulses were used.

**Fig 2:**
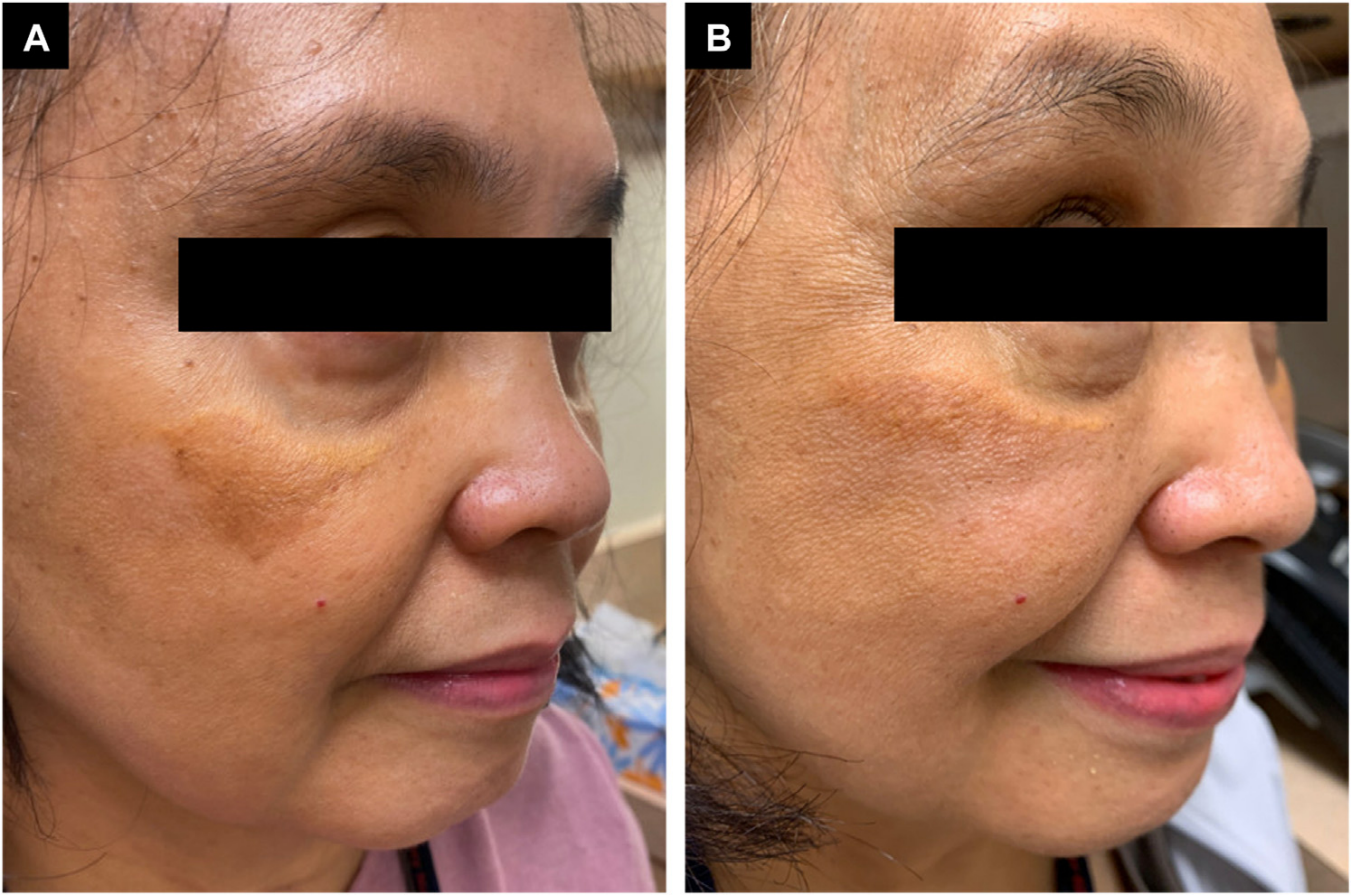
Improvement in *yellow*-*brown* infraorbital xanthogranulomatous (XG) plaque following 1 treatment after treatment with fractional carbon dioxide (CO_2_) laser. (**A**) Before treatment (**B**) 26 months after 1 treatment.

The patient returned to clinic in 2023 with significant improvement of the XG plaque on her right cheek following 1 treatment with fractional CO_2_ laser. A marked improvement in hyperpigmentation along the inferior aspect of the lesion was observed, along with improvement in the yellow appearance of the plaque with mild hypopigmentation (Fig 2). Some lateral extension of the plaque is observed, which may be attributable to a 2-year interval between the second laser treatment and subsequent evaluation with no additional therapies used during that time.

## Discussion

Treatment of periorbital xanthogranulomas can be difficult. One study found that patients who underwent surgical excision of NXG lesions developed xanthogranulomas at the site of removal 6 to 12 months later.^2^ Medical management of NXG with intravenous immunoglobulin, corticosteroids, interferon alpha, alkylating agents, antimetabolites, cladribine, and phototherapy are often used but with variable efficacy.^3^ The use of various types of lasers in the treatment of XG disorders has been discussed in the literature but are less utilized.

One case report described a patient with plane xanthoma who was previously treated with pulsed dye laser (595 nm) and alexandrite laser (755 nm) without effect but saw improvement with the use of quality-switched neodymium-doped yttrium aluminum garnet laser filtered to 650 nm.^4^ Wee et al presented a case of a patient with facial NXG treated with neodymium-doped yttrium aluminum garnet laser (1064 nm) without improvement.^5^ Meanwhile, ablative CO_2_ lasers have been shown to be efficacious in cases of NXG and juvenile xanthogranuloma but were limited by scarring and pain.^6^ However, fractional CO_2_ lasers have been effective in treatment of NXG and juvenile xanthogranuloma^7^ with reduced morbidity compared to fully ablative lasers.

Lasers used in the treatment of skin conditions operate under the principle of selective photothermolysis – the concept that specific wavelengths of light can be selectively absorbed by a specific chromophore in the skin and subsequently destroyed with minimal damage to surrounding tissue. Fractional lasers take this concept 1 step further with fractional photothermolysis where the same targeted destruction occurs but is limited to an array of microscopic columns in the skin called micro thermal zones. Histopathologic studies have shown that thermal damage caused by the micro thermal zones extend to a depth of 300 to 400 μm into the dermis, stimulating wound healing with collagen remodeling and elastin deposition, lasting at least 3 months following treatment.^8^

The wound healing promoted by fractional photothermolysis may explain the efficacy observed in fractional CO_2_ laser treatments in XG disorders. Kersai et al described the use of fractional photothermolysis in the treatment of granuloma annulare. It was observed histologically that the areas of the skin at 3 weeks post-treatment showed clearance of granulomas.^9^ While the exact mechanism is not fully understood, it is theorized that the activation of nonspecific wound healing via fractional photothermolysis stimulates the release of prostaglandin E2 from neighboring macrophages, thus inhibiting granuloma formation, which can potentially help in treatment of granulomatous diseases, including NXG.

Rarely, instances of exacerbation and ulceration of granulomatous disease with laser treatment has been noted in the literature. In 1 case, actively inflamed lupus pernio treated with flashlamp-pumped pulsed dye laser resulted in ulceration of existing and development of new sarcoid lesions.^10^ Laser treatment in patients with active granulomatous inflammation should be approached with caution. Additionally, hypopigmentation is a possible adverse outcome in patients with darker phototypes, as demonstrated by our patient, and should be considered when discussing treatment options.

In patients with periborbital xanthogranulomas, the presence of facial lesions can be distressing and difficult to treat. This case of refractory periorbital xanthogranulomas successfully treated with fractional CO_2_ laser demonstrates that fractional photothermolysis can be a viable treatment option.

## Conflicts of interest

None disclosed.
